# Ethyl (*E*)-3-hy­droxy-2-{*N*-[2-(thio­phen-2-yl)ethen­yl]carbamo­yl}but-2-enoate

**DOI:** 10.1107/S1600536812028176

**Published:** 2012-06-30

**Authors:** Bao-Shuo Liu, Sheng-Yin Zhao

**Affiliations:** a College of Chemistry, Chemical Engineering and Biotechnology, Donghua University, Shanghai 201620, People’s Republic of China

## Abstract

In the title compound, C_13_H_15_NO_4_S, there are two independent but conformationally similar mol­ecules in the asymmetric unit, both having an *E* conformation of the side-chain C=C group. Intra­molecular N—H⋯O and O—H⋯O hydrogen-bonding inter­actions are present in both molecules. In the crystal, one of the mol­ecule types is linked through inter­molecular hy­droxy–ketone O—H⋯O inter­actions, forming one-dimensional chains extending along [010], whereas the other mol­ecule type shows no associations.

## Related literature

For applications of 4-hy­droxy-2-pyridones, see: Buisson *et al.* (1996 ▶); Jessen & Gademann (2010 ▶). For general background to the synthesis, see: Rigby & Burkhardt (1986 ▶); Rigby & Qabar (1989 ▶). For the structure of a similar compound, see: Zhao & Huang (2012 ▶).
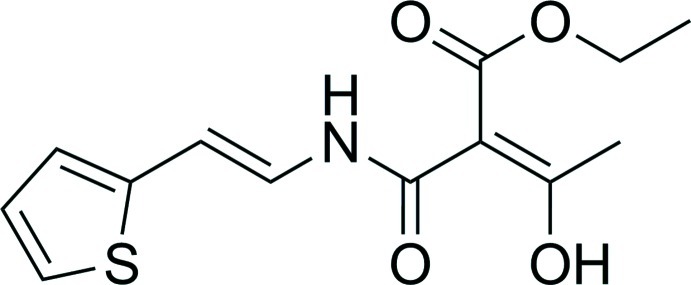


## Experimental

### 

#### Crystal data

C_13_H_15_NO_4_S
*M* *_r_* = 281.33
Monoclinic, 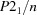

*a* = 14.0185 (15) Å
*b* = 13.1232 (14) Å
*c* = 15.0141 (16) Å
β = 96.853 (2)°
*V* = 2742.4 (5) Å^3^
*Z* = 8
Mo *K*α radiation
μ = 0.25 mm^−1^
*T* = 293 K
0.32 × 0.21 × 0.15 mm

#### Data collection

Bruker SMART CCD area-detector diffractometer
Absorption correction: multi-scan (*SADABS*; Bruker, 2003 ▶) *T*_min_ = 0.394, *T*_max_ = 1.000
15596 measured reflections
5099 independent reflections
3538 reflections with *I* > 2σ(*I*)
*R*_int_ = 0.061

#### Refinement

*R*[*F* ^2^ > 2σ(*F* ^2^)] = 0.057
*wR*(*F* ^2^) = 0.179
*S* = 1.05
5099 reflections
357 parameters
1 restraint
H atoms treated by a mixture of independent and constrained refinement
Δρ_max_ = 0.42 e Å^−3^
Δρ_min_ = −0.34 e Å^−3^

### 

Data collection: *SMART* (Bruker, 2003 ▶); cell refinement: *SAINT* (Bruker, 2003 ▶); data reduction: *SAINT*; program(s) used to solve structure: *SHELXS97* (Sheldrick, 2008 ▶); program(s) used to refine structure: *SHELXL97* (Sheldrick, 2008 ▶); molecular graphics: *SHELXTL* (Sheldrick, 2008 ▶); software used to prepare material for publication: *SHELXTL*.

## Supplementary Material

Crystal structure: contains datablock(s) I, global. DOI: 10.1107/S1600536812028176/zs2216sup1.cif

Structure factors: contains datablock(s) I. DOI: 10.1107/S1600536812028176/zs2216Isup2.hkl

Supplementary material file. DOI: 10.1107/S1600536812028176/zs2216Isup3.cml

Additional supplementary materials:  crystallographic information; 3D view; checkCIF report

## Figures and Tables

**Table 1:** Hydrogen-bond geometry (Å, °)

*D*—H⋯*A*	*D*—H	H⋯*A*	*D*⋯*A*	*D*—H⋯*A*
N1′—H1′*A*⋯O3′	0.77 (3)	1.98 (3)	2.615 (3)	139 (3)
N1—H1*A*⋯O3	0.68 (3)	2.10 (3)	2.637 (3)	137 (3)
O2′—H2′⋯O1′	0.82	1.65	2.399 (3)	152
O2—H2*A*⋯O1	0.82	1.67	2.419 (3)	151
O2—H2*A*⋯O3^i^	0.82	2.48	2.936 (3)	117

Symmetry code: (i) 
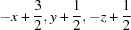
.

**Fig. 1.**
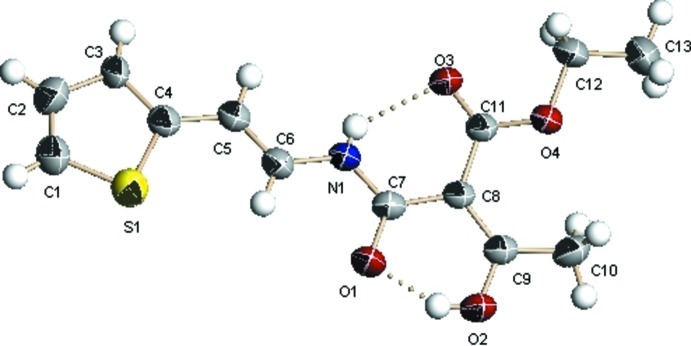
The atom-numbering scheme of the first molecule in the asymmetric unit of the title compound, with displacement ellipsoids drawn at the 30% probability level. Hydrogen bonds are shown as dashed lines.

**Fig. 2.**
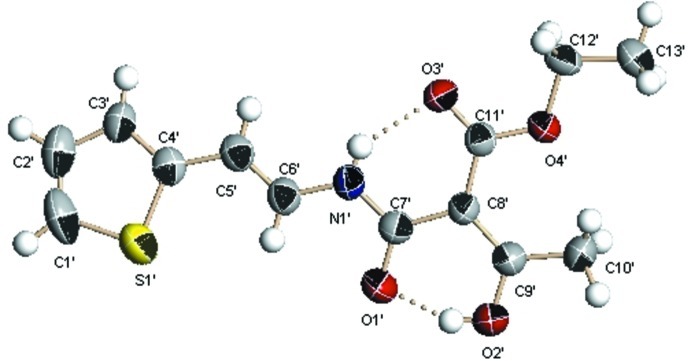
The atom-numbering scheme of the second molecule in the asymmetric unit of the title compound.

**Fig. 3.**
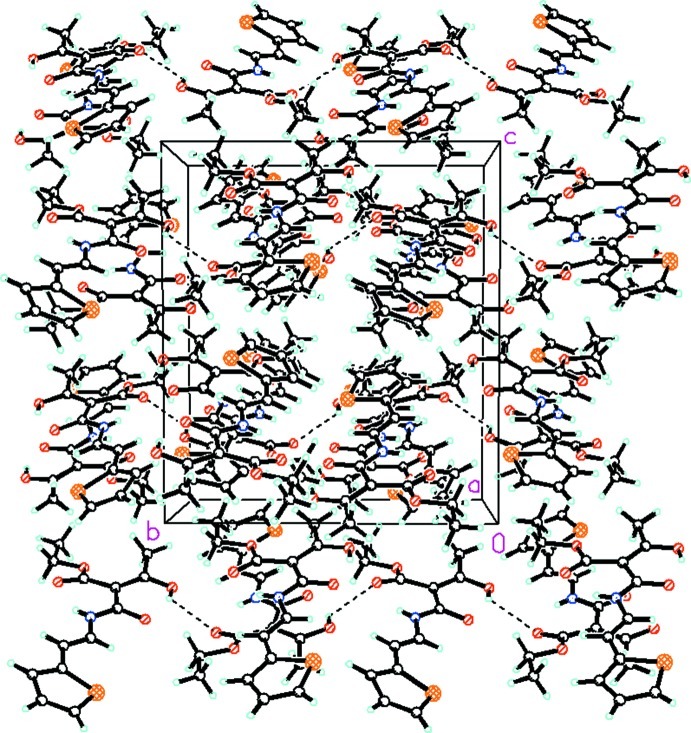
Molecular packing of the title compound viewed down the a axis of the unit cell, with O—H···O interactions shown as dashed lines.

## supplementary crystallographic information

### Comment

The derivatives of 4-hydroxy-2-pyridones exhibit a wide range of biological activities (Buisson *et al.*, 1996; Jessen & Gademann, 2010). The title compound, C_13_H_15_NO_4_S, is an important intermediate in the synthesis of 4-hydroxy-2-pyridone derivatives (Rigby & Burkhardt, 1986; Rigby & Qabar, 1989). In the title compound, there are two independent but conformationally similar molecules in the asymmetric unit (Figs. 1 and 2), both of which have an *E* configuration of the side chain C5═C6. The molecular conformation is stabilized by intramolecular N—H···O_ketone_ and hydroxyl O—H···O_ketone_ hydrogen bonds (Table 1, Figs. 1 and 2).

In the crystal, only one of the molecule types is linked through intermolecular hydroxyl O—H···O_ketone_ interactions forming one-dimensional chain structures extending along (010) (Fig. 3), whereas the other molecule type is unassociated. The thiophene rings have normal hydrophobic contacts without any stacking interactions. For the structure of a similar compound, see Zhao & Huang (2012).

### Experimental

To an ice-cooled solution of 3-(2-thienyl)acrylic acid (5.0 g, 32.5 mmol) in 70 ml of ethyl acetate was added triethylamine (4.3 g, 42.2 mmol) and diphenyl phosphorazidate (DPPA, 11.6 g, 42.2 mmol). The solution was stirred at room temperature for 4 h. The acyl azide product was washed by dilution with cold water. The organic layers were dried over MgSO_4_, and the solvent was removed under reduced pressure (< 318 K). The acyl azide was dissolved in 50 ml of benzene and heated under reflux until azide decomposition was complete. The reaction mixture was then cooled to 273 K and ethyl sodio-acetoacetate [prepared from ethyl acetoacetate (5.07 g, 39.0 mmol) and sodium hydride (1.1 g, 60% dispersion in oil, 45.5 mmol) in toluene (100 ml) at 273 K] was added. After warming to room temperature for 2 h, the mixture was quenched with saturated aqueous ammonium chloride solution, rinsed with brine, and dried over MgSO_4_. The solvent was removed *in vacuo* to give green crystals: 5.57 g, yield, 61.0% (m.p. 366–368 K). 1H NMR (400 MHz, chloroform-d) 1.37 (t, J = 7.1 Hz, 3H, CH_3_), 2.47 (s, 3H, CH_3_), 4.29 (q, J = 7.1 Hz, 2H, CH_2_), 6.41 (d, J = 14.5 Hz, 1H, =CH), 6.91 (s, 1H, Ar—H), 6.93 (d, J = 4.4 Hz, 1H, Ar—H), 7.10 (d, J = 4.9 Hz, 1H, Ar—H), 7.41 (dd, J = 14.3, 10.8 Hz, 1H, =CH), 11.06 (d, J = 10.0 Hz, 1H, NH), 18.04 (s, 1H, OH). Crystals suitable for single-crystal X-ray diffraction were grown by slow evaporation at room temperature from a solution in a mixture of hexane and ethyl acetate (10:1).

### Refinement

The amine hydrogen atom was located in a difference-Fourier map and refined freely. Other hydrogen atoms were positioned geometrically and refined using a riding model with O—H = 0.82 Å, C—H = 0.93 Å (CH), 0.96 Å (CH_3_) or 0.97 Å (CH_2_). Isotropic displacement parameters for these atoms were set to 1.2 (CH, CH_2_) or 1.5 (OH, CH_3_) times *U*_eq_ of the parent atom.

### Crystal data

**Table:**

C_13_H_15_NO_4_S	*F*(000) = 1184
*M**_r_* = 281.33	*D*_x_ = 1.363 Mg m^−^^3^
Monoclinic, *P*2_1_/*n*	Melting point = 366–368 K
Hall symbol: -P 2yn	Mo *K*α radiation, λ = 0.71073 Å
*a* = 14.0185 (15) Å	Cell parameters from 3746 reflections
*b* = 13.1232 (14) Å	θ = 4.9–26.5°
*c* = 15.0141 (16) Å	µ = 0.25 mm^−^^1^
β = 96.853 (2)°	*T* = 293 K
*V* = 2742.4 (5) Å^3^	Prismatic, white
*Z* = 8	0.32 × 0.21 × 0.15 mm

### Data collection

**Table:**

Bruker SMART CCD area-detector diffractometer	5099 independent reflections
Radiation source: fine-focus sealed tube	3538 reflections with *I* > 2σ(*I*)
Graphite monochromator	*R*_int_ = 0.061
φ and ω scans	θ_max_ = 25.5°, θ_min_ = 2.1°
Absorption correction: multi-scan (*SADABS*; Bruker, 2003)	*h* = −15→16
*T*_min_ = 0.394, *T*_max_ = 1.000	*k* = −15→14
15596 measured reflections	*l* = −18→17

### Refinement

**Table:**

Refinement on *F*^2^	Primary atom site location: structure-invariant direct methods
Least-squares matrix: full	Secondary atom site location: difference Fourier map
*R*[*F*^2^ > 2σ(*F*^2^)] = 0.057	Hydrogen site location: inferred from neighbouring sites
*wR*(*F*^2^) = 0.179	H atoms treated by a mixture of independent and constrained refinement
*S* = 1.05	*w* = 1/[σ^2^(*F*_o_^2^) + (0.1017*P*)^2^ + 0.3594*P*] where *P* = (*F*_o_^2^ + 2*F*_c_^2^)/3
5099 reflections	(Δ/σ)_max_ < 0.001
357 parameters	Δρ_max_ = 0.42 e Å^−^^3^
1 restraint	Δρ_min_ = −0.34 e Å^−^^3^

### Special details

**Table:**

Geometry. All e.s.d.'s (except the e.s.d. in the dihedral angle between two l.s. planes) are estimated using the full covariance matrix. The cell e.s.d.'s are taken into account individually in the estimation of e.s.d.'s in distances, angles and torsion angles; correlations between e.s.d.'s in cell parameters are only used when they are defined by crystal symmetry. An approximate (isotropic) treatment of cell e.s.d.'s is used for estimating e.s.d.'s involving l.s. planes.
Refinement. Refinement of *F*^2^ against ALL reflections. The weighted *R*-factor *wR* and goodness of fit *S* are based on *F*^2^, conventional *R*-factors *R* are based on *F*, with *F* set to zero for negative *F*^2^. The threshold expression of *F*^2^ > σ(*F*^2^) is used only for calculating *R*-factors(gt) *etc*. and is not relevant to the choice of reflections for refinement. *R*-factors based on *F*^2^ are statistically about twice as large as those based on *F*, and *R*- factors based on ALL data will be even larger.

### Fractional atomic coordinates and isotropic or equivalent isotropic displacement parameters (Å^2^)

**Table:**

	*x*	*y*	*z*	*U*_iso_*/*U*_eq_	
S1	0.36218 (6)	0.30430 (6)	0.06353 (5)	0.0674 (3)	
S1'	0.60812 (7)	0.54606 (7)	0.67909 (7)	0.0873 (3)	
N1	0.64877 (16)	0.25510 (18)	0.23340 (16)	0.0511 (6)	
N1'	0.89786 (17)	0.6539 (2)	0.81282 (16)	0.0570 (6)	
O1	0.68462 (15)	0.41967 (13)	0.22337 (15)	0.0716 (6)	
O2	0.83238 (15)	0.49138 (13)	0.29706 (14)	0.0659 (6)	
H2A	0.7772	0.4877	0.2721	0.099*	
O3	0.78402 (14)	0.13804 (13)	0.31666 (15)	0.0682 (6)	
O4	0.93063 (13)	0.20136 (12)	0.34348 (13)	0.0594 (5)	
O1'	0.94002 (16)	0.49320 (16)	0.84332 (16)	0.0792 (7)	
O2'	1.09020 (16)	0.44802 (15)	0.92784 (17)	0.0805 (7)	
H2'	1.0350	0.4438	0.9029	0.121*	
O3'	1.02266 (15)	0.79503 (14)	0.86837 (14)	0.0699 (6)	
O4'	1.16883 (13)	0.74930 (13)	0.92788 (12)	0.0583 (5)	
C1	0.2546 (2)	0.2520 (2)	0.02830 (19)	0.0658 (8)	
H1	0.2065	0.2853	−0.0086	0.079*	
C2	0.2461 (2)	0.1575 (2)	0.0591 (2)	0.0702 (8)	
H2	0.1909	0.1187	0.0447	0.084*	
C3	0.32914 (19)	0.1194 (2)	0.11653 (18)	0.0571 (7)	
H3	0.3346	0.0561	0.1444	0.068*	
C4	0.40153 (18)	0.19769 (18)	0.12237 (17)	0.0489 (6)	
C5	0.49619 (18)	0.19051 (18)	0.17316 (17)	0.0512 (6)	
H5	0.5141	0.1279	0.1989	0.061*	
C6	0.55842 (19)	0.26513 (19)	0.18571 (17)	0.0503 (6)	
H6	0.5409	0.3285	0.1612	0.060*	
C7	0.71066 (19)	0.33179 (17)	0.25083 (17)	0.0492 (6)	
C8	0.80519 (18)	0.31539 (16)	0.29962 (16)	0.0442 (6)	
C9	0.86181 (18)	0.40078 (18)	0.32367 (17)	0.0483 (6)	
C10	0.9549 (2)	0.4027 (2)	0.3815 (2)	0.0631 (8)	
H10A	1.0050	0.3813	0.3474	0.095*	
H10B	0.9522	0.3574	0.4313	0.095*	
H10C	0.9678	0.4707	0.4032	0.095*	
C11	0.83634 (18)	0.21138 (17)	0.32113 (16)	0.0470 (6)	
C12	0.9659 (2)	0.0986 (2)	0.3595 (2)	0.0732 (9)	
H12A	0.9616	0.0610	0.3035	0.088*	
H12B	0.9279	0.0634	0.3998	0.088*	
C13	1.0655 (3)	0.1052 (3)	0.3993 (3)	0.1086 (15)	
H13A	1.1034	0.1357	0.3572	0.163*	
H13B	1.0894	0.0381	0.4145	0.163*	
H13C	1.0694	0.1463	0.4526	0.163*	
C1'	0.5056 (3)	0.5833 (4)	0.6165 (3)	0.0967 (13)	
H1'	0.4553	0.5394	0.5973	0.116*	
C2'	0.5052 (2)	0.6825 (4)	0.5977 (2)	0.0845 (11)	
H2'1	0.4546	0.7156	0.5637	0.101*	
C3'	0.5891 (2)	0.7312 (3)	0.63484 (19)	0.0637 (7)	
H3'	0.6001	0.8005	0.6283	0.076*	
C4'	0.6534 (2)	0.6673 (2)	0.68161 (17)	0.0557 (7)	
C5'	0.7466 (2)	0.6926 (2)	0.72761 (18)	0.0576 (7)	
H5'	0.7641	0.7610	0.7286	0.069*	
C6'	0.8093 (2)	0.6281 (2)	0.76828 (18)	0.0576 (7)	
H6'	0.7925	0.5595	0.7669	0.069*	
C7'	0.9618 (2)	0.5860 (2)	0.84934 (18)	0.0556 (7)	
C8'	1.05502 (19)	0.61925 (19)	0.89353 (17)	0.0501 (6)	
C9'	1.1163 (2)	0.5435 (2)	0.93281 (19)	0.0594 (7)	
C10'	1.2130 (2)	0.5563 (2)	0.9827 (2)	0.0771 (9)	
H10D	1.2297	0.4963	1.0177	0.116*	
H10E	1.2130	0.6143	1.0218	0.116*	
H10F	1.2590	0.5667	0.9411	0.116*	
C11'	1.07879 (19)	0.7279 (2)	0.89515 (16)	0.0505 (6)	
C12'	1.1946 (2)	0.8560 (2)	0.93642 (19)	0.0609 (7)	
H12C	1.1504	0.8917	0.9706	0.073*	
H12D	1.1917	0.8873	0.8776	0.073*	
C13'	1.2938 (2)	0.8616 (2)	0.9834 (2)	0.0691 (8)	
H13D	1.2952	0.8330	1.0423	0.104*	
H13E	1.3140	0.9315	0.9880	0.104*	
H13F	1.3364	0.8240	0.9501	0.104*	
H1A	0.661 (2)	0.208 (2)	0.2506 (19)	0.055 (9)*	
H1'A	0.912 (2)	0.711 (2)	0.818 (2)	0.066 (10)*	

### Atomic displacement parameters (Å^2^)

**Table:**

	*U*^11^	*U*^22^	*U*^33^	*U*^12^	*U*^13^	*U*^23^
S1	0.0635 (5)	0.0618 (5)	0.0737 (5)	−0.0007 (3)	−0.0051 (4)	0.0088 (3)
S1'	0.0765 (6)	0.0780 (6)	0.1028 (7)	−0.0186 (5)	−0.0078 (5)	0.0014 (5)
N1	0.0471 (13)	0.0350 (12)	0.0689 (15)	0.0038 (10)	−0.0025 (10)	0.0029 (10)
N1'	0.0489 (14)	0.0548 (15)	0.0644 (15)	0.0007 (11)	−0.0052 (11)	−0.0071 (11)
O1	0.0608 (13)	0.0382 (10)	0.1093 (16)	0.0041 (9)	−0.0166 (11)	0.0125 (10)
O2	0.0665 (14)	0.0344 (9)	0.0914 (15)	−0.0025 (8)	−0.0122 (11)	0.0027 (9)
O3	0.0576 (12)	0.0332 (9)	0.1070 (16)	−0.0027 (8)	−0.0186 (11)	0.0007 (9)
O4	0.0492 (11)	0.0376 (9)	0.0874 (13)	0.0054 (8)	−0.0084 (10)	−0.0022 (8)
O1'	0.0706 (14)	0.0529 (12)	0.1078 (17)	−0.0076 (10)	−0.0155 (12)	−0.0068 (11)
O2'	0.0668 (15)	0.0505 (12)	0.1185 (19)	0.0000 (10)	−0.0117 (13)	0.0073 (11)
O3'	0.0685 (14)	0.0478 (10)	0.0869 (14)	0.0034 (9)	−0.0175 (11)	0.0026 (9)
O4'	0.0521 (11)	0.0458 (10)	0.0752 (12)	−0.0046 (8)	−0.0004 (9)	−0.0048 (8)
C1	0.0579 (18)	0.0688 (19)	0.0661 (18)	0.0031 (14)	−0.0113 (14)	−0.0008 (14)
C2	0.0564 (18)	0.0648 (18)	0.083 (2)	−0.0062 (14)	−0.0199 (14)	−0.0006 (15)
C3	0.0506 (15)	0.0484 (14)	0.0669 (17)	0.0010 (12)	−0.0149 (12)	−0.0134 (12)
C4	0.0504 (15)	0.0441 (13)	0.0510 (14)	0.0044 (11)	0.0007 (12)	−0.0056 (10)
C5	0.0494 (15)	0.0413 (13)	0.0615 (16)	0.0053 (11)	0.0007 (12)	−0.0027 (11)
C6	0.0489 (15)	0.0428 (13)	0.0575 (15)	0.0068 (11)	−0.0009 (12)	0.0005 (11)
C7	0.0515 (16)	0.0361 (12)	0.0592 (15)	0.0015 (11)	0.0036 (12)	−0.0002 (10)
C8	0.0481 (15)	0.0336 (12)	0.0498 (13)	0.0017 (10)	0.0017 (11)	−0.0035 (9)
C9	0.0529 (16)	0.0374 (13)	0.0541 (15)	−0.0004 (11)	0.0042 (12)	−0.0021 (10)
C10	0.0678 (19)	0.0431 (14)	0.0742 (18)	−0.0095 (13)	−0.0092 (15)	−0.0037 (12)
C11	0.0494 (15)	0.0362 (12)	0.0526 (14)	0.0016 (11)	−0.0051 (11)	−0.0044 (10)
C12	0.0647 (19)	0.0408 (15)	0.107 (2)	0.0112 (13)	−0.0169 (17)	−0.0062 (14)
C13	0.068 (2)	0.067 (2)	0.179 (4)	0.0143 (18)	−0.034 (3)	−0.007 (2)
C1'	0.065 (2)	0.137 (4)	0.085 (2)	−0.029 (2)	−0.0066 (18)	−0.025 (2)
C2'	0.058 (2)	0.125 (3)	0.067 (2)	0.008 (2)	−0.0069 (16)	−0.008 (2)
C3'	0.0518 (17)	0.0746 (19)	0.0628 (17)	0.0080 (14)	−0.0007 (13)	−0.0031 (14)
C4'	0.0528 (17)	0.0661 (17)	0.0484 (15)	−0.0024 (13)	0.0063 (12)	−0.0088 (12)
C5'	0.0533 (17)	0.0620 (16)	0.0573 (16)	−0.0024 (13)	0.0055 (13)	−0.0075 (13)
C6'	0.0547 (17)	0.0616 (16)	0.0548 (16)	−0.0010 (13)	−0.0008 (13)	−0.0072 (12)
C7'	0.0558 (17)	0.0484 (15)	0.0612 (17)	0.0003 (12)	0.0021 (13)	−0.0056 (12)
C8'	0.0464 (15)	0.0470 (13)	0.0560 (15)	0.0023 (11)	0.0020 (12)	−0.0026 (11)
C9'	0.0541 (17)	0.0515 (16)	0.0710 (18)	0.0005 (12)	0.0014 (14)	0.0016 (13)
C10'	0.0569 (19)	0.0627 (18)	0.106 (3)	0.0054 (15)	−0.0114 (17)	0.0150 (17)
C11'	0.0514 (16)	0.0523 (14)	0.0464 (14)	−0.0004 (12)	0.0001 (12)	−0.0022 (11)
C12'	0.0695 (19)	0.0465 (15)	0.0656 (17)	−0.0104 (13)	0.0035 (14)	−0.0012 (12)
C13'	0.0647 (19)	0.0686 (19)	0.073 (2)	−0.0158 (15)	0.0051 (15)	−0.0085 (15)

### Geometric parameters (Å, º)

**Table:**

S1—C1	1.684 (3)	C8—C11	1.458 (3)
S1—C4	1.710 (3)	C9—C10	1.478 (3)
S1'—C1'	1.692 (4)	C10—H10A	0.9600
S1'—C4'	1.712 (3)	C10—H10B	0.9600
N1—C7	1.334 (3)	C10—H10C	0.9600
N1—C6	1.385 (3)	C12—C13	1.454 (4)
N1—H1A	0.68 (3)	C12—H12A	0.9700
N1'—C7'	1.335 (4)	C12—H12B	0.9700
N1'—C6'	1.381 (3)	C13—H13A	0.9600
N1'—H1'A	0.77 (3)	C13—H13B	0.9600
O1—C7	1.264 (3)	C13—H13C	0.9600
O2—C9	1.305 (3)	C1'—C2'	1.332 (6)
O2—H2A	0.8200	C1'—H1'	0.9300
O3—C11	1.207 (3)	C2'—C3'	1.396 (4)
O4—C11	1.331 (3)	C2'—H2'1	0.9300
O4—C12	1.447 (3)	C3'—C4'	1.363 (4)
O1'—C7'	1.256 (3)	C3'—H3'	0.9300
O2'—C9'	1.304 (3)	C4'—C5'	1.442 (4)
O2'—H2'	0.8200	C5'—C6'	1.316 (4)
O3'—C11'	1.217 (3)	C5'—H5'	0.9300
O4'—C11'	1.329 (3)	C6'—H6'	0.9300
O4'—C12'	1.448 (3)	C7'—C8'	1.460 (4)
C1—C2	1.334 (4)	C8'—C9'	1.398 (4)
C1—H1	0.9300	C8'—C11'	1.464 (4)
C2—C3	1.452 (4)	C9'—C10'	1.478 (4)
C2—H2	0.9300	C10'—H10D	0.9600
C3—C4	1.439 (4)	C10'—H10E	0.9600
C3—H3	0.9300	C10'—H10F	0.9600
C4—C5	1.453 (3)	C12'—C13'	1.484 (4)
C5—C6	1.310 (4)	C12'—H12C	0.9700
C5—H5	0.9300	C12'—H12D	0.9700
C6—H6	0.9300	C13'—H13D	0.9600
C7—C8	1.452 (3)	C13'—H13E	0.9600
C8—C9	1.396 (3)	C13'—H13F	0.9600
			
C1—S1—C4	92.87 (14)	C12—C13—H13A	109.5
C1'—S1'—C4'	91.8 (2)	C12—C13—H13B	109.5
C7—N1—C6	124.3 (2)	H13A—C13—H13B	109.5
C7—N1—H1A	119 (3)	C12—C13—H13C	109.5
C6—N1—H1A	117 (3)	H13A—C13—H13C	109.5
C7'—N1'—C6'	123.8 (3)	H13B—C13—H13C	109.5
C7'—N1'—H1'A	117 (2)	C2'—C1'—S1'	112.5 (3)
C6'—N1'—H1'A	120 (2)	C2'—C1'—H1'	123.8
C9—O2—H2A	109.5	S1'—C1'—H1'	123.8
C11—O4—C12	116.3 (2)	C1'—C2'—C3'	112.3 (3)
C9'—O2'—H2'	109.5	C1'—C2'—H2'1	123.8
C11'—O4'—C12'	117.0 (2)	C3'—C2'—H2'1	123.8
C2—C1—S1	112.6 (2)	C4'—C3'—C2'	113.5 (3)
C2—C1—H1	123.7	C4'—C3'—H3'	123.3
S1—C1—H1	123.7	C2'—C3'—H3'	123.3
C1—C2—C3	115.3 (3)	C3'—C4'—C5'	127.6 (3)
C1—C2—H2	122.4	C3'—C4'—S1'	109.9 (2)
C3—C2—H2	122.4	C5'—C4'—S1'	122.5 (2)
C4—C3—C2	107.5 (2)	C6'—C5'—C4'	126.2 (3)
C4—C3—H3	126.3	C6'—C5'—H5'	116.9
C2—C3—H3	126.3	C4'—C5'—H5'	116.9
C3—C4—C5	125.4 (2)	C5'—C6'—N1'	125.3 (3)
C3—C4—S1	111.76 (19)	C5'—C6'—H6'	117.3
C5—C4—S1	122.84 (19)	N1'—C6'—H6'	117.3
C6—C5—C4	125.3 (2)	O1'—C7'—N1'	118.2 (3)
C6—C5—H5	117.3	O1'—C7'—C8'	121.3 (2)
C4—C5—H5	117.3	N1'—C7'—C8'	120.5 (2)
C5—C6—N1	123.8 (2)	C9'—C8'—C7'	116.8 (2)
C5—C6—H6	118.1	C9'—C8'—C11'	124.0 (2)
N1—C6—H6	118.1	C7'—C8'—C11'	119.2 (2)
O1—C7—N1	117.9 (2)	O2'—C9'—C8'	120.3 (3)
O1—C7—C8	120.7 (2)	O2'—C9'—C10'	111.9 (2)
N1—C7—C8	121.3 (2)	C8'—C9'—C10'	127.8 (3)
C9—C8—C7	117.9 (2)	C9'—C10'—H10D	109.5
C9—C8—C11	123.3 (2)	C9'—C10'—H10E	109.5
C7—C8—C11	118.8 (2)	H10D—C10'—H10E	109.5
O2—C9—C8	120.2 (2)	C9'—C10'—H10F	109.5
O2—C9—C10	112.8 (2)	H10D—C10'—H10F	109.5
C8—C9—C10	126.9 (2)	H10E—C10'—H10F	109.5
C9—C10—H10A	109.5	O3'—C11'—O4'	121.1 (2)
C9—C10—H10B	109.5	O3'—C11'—C8'	124.2 (2)
H10A—C10—H10B	109.5	O4'—C11'—C8'	114.6 (2)
C9—C10—H10C	109.5	O4'—C12'—C13'	107.5 (2)
H10A—C10—H10C	109.5	O4'—C12'—H12C	110.2
H10B—C10—H10C	109.5	C13'—C12'—H12C	110.2
O3—C11—O4	120.9 (2)	O4'—C12'—H12D	110.2
O3—C11—C8	124.8 (2)	C13'—C12'—H12D	110.2
O4—C11—C8	114.2 (2)	H12C—C12'—H12D	108.5
O4—C12—C13	107.8 (2)	C12'—C13'—H13D	109.5
O4—C12—H12A	110.2	C12'—C13'—H13E	109.5
C13—C12—H12A	110.2	H13D—C13'—H13E	109.5
O4—C12—H12B	110.2	C12'—C13'—H13F	109.5
C13—C12—H12B	110.2	H13D—C13'—H13F	109.5
H12A—C12—H12B	108.5	H13E—C13'—H13F	109.5
			
C4—S1—C1—C2	0.3 (3)	C4'—S1'—C1'—C2'	0.3 (3)
S1—C1—C2—C3	0.6 (4)	S1'—C1'—C2'—C3'	−0.4 (4)
C1—C2—C3—C4	−1.4 (4)	C1'—C2'—C3'—C4'	0.4 (4)
C2—C3—C4—C5	−179.8 (3)	C2'—C3'—C4'—C5'	179.7 (3)
C2—C3—C4—S1	1.6 (3)	C2'—C3'—C4'—S1'	−0.2 (3)
C1—S1—C4—C3	−1.2 (2)	C1'—S1'—C4'—C3'	−0.1 (2)
C1—S1—C4—C5	−179.8 (2)	C1'—S1'—C4'—C5'	−179.9 (2)
C3—C4—C5—C6	−172.9 (3)	C3'—C4'—C5'—C6'	−176.2 (3)
S1—C4—C5—C6	5.6 (4)	S1'—C4'—C5'—C6'	3.7 (4)
C4—C5—C6—N1	−178.9 (2)	C4'—C5'—C6'—N1'	−179.3 (3)
C7—N1—C6—C5	−176.1 (3)	C7'—N1'—C6'—C5'	−176.3 (3)
C6—N1—C7—O1	1.2 (4)	C6'—N1'—C7'—O1'	−0.4 (4)
C6—N1—C7—C8	−178.6 (2)	C6'—N1'—C7'—C8'	178.5 (3)
O1—C7—C8—C9	6.9 (4)	O1'—C7'—C8'—C9'	−3.4 (4)
N1—C7—C8—C9	−173.2 (2)	N1'—C7'—C8'—C9'	177.8 (3)
O1—C7—C8—C11	−173.1 (2)	O1'—C7'—C8'—C11'	177.1 (3)
N1—C7—C8—C11	6.7 (4)	N1'—C7'—C8'—C11'	−1.7 (4)
C7—C8—C9—O2	−4.7 (4)	C7'—C8'—C9'—O2'	1.1 (4)
C11—C8—C9—O2	175.3 (2)	C11'—C8'—C9'—O2'	−179.4 (3)
C7—C8—C9—C10	172.8 (3)	C7'—C8'—C9'—C10'	−178.8 (3)
C11—C8—C9—C10	−7.2 (4)	C11'—C8'—C9'—C10'	0.7 (5)
C12—O4—C11—O3	1.5 (4)	C12'—O4'—C11'—O3'	4.6 (4)
C12—O4—C11—C8	−175.9 (2)	C12'—O4'—C11'—C8'	−176.1 (2)
C9—C8—C11—O3	165.5 (3)	C9'—C8'—C11'—O3'	−173.0 (3)
C7—C8—C11—O3	−14.5 (4)	C7'—C8'—C11'—O3'	6.4 (4)
C9—C8—C11—O4	−17.2 (4)	C9'—C8'—C11'—O4'	7.7 (4)
C7—C8—C11—O4	162.8 (2)	C7'—C8'—C11'—O4'	−172.8 (2)
C11—O4—C12—C13	−169.1 (3)	C11'—O4'—C12'—C13'	173.7 (2)

### Hydrogen-bond geometry (Å, º)

**Table:**

*D*—H···*A*	*D*—H	H···*A*	*D*···*A*	*D*—H···*A*
N1′—H1′*A*···O3′	0.77 (3)	1.98 (3)	2.615 (3)	139 (3)
N1—H1*A*···O3	0.68 (3)	2.10 (3)	2.637 (3)	137 (3)
O2′—H2′···O1′	0.82	1.65	2.399 (3)	152
O2—H2*A*···O1	0.82	1.67	2.419 (3)	151
O2—H2*A*···O3^i^	0.82	2.48	2.936 (3)	117

Symmetry code: (i) −*x*+3/2, *y*+1/2, −*z*+1/2.
